# Assessment of a novel multiplex real-time PCR assay for the detection of the CBPP agent *Mycoplasma mycoides *subsp. *mycoides *SC through experimental infection in cattle

**DOI:** 10.1186/1746-6148-7-47

**Published:** 2011-08-12

**Authors:** Christiane Schnee, Martin Heller, Jörg Jores, Herbert Tomaso, Heinrich Neubauer

**Affiliations:** 1 Institute of Bacterial Infections and Zoonoses, Friedrich-Loeffler-Institute, Federal Research Institute for Animal Health, Naumburger Strasse 96a, 07743 Jena, Germany; 2 International Livestock Research Institute, Old Naivasha Road, P.O. Box 30709, 00100 Nairobi, Kenya

## Abstract

**Background:**

*Mycoplasma mycoides *subsp. *mycoides *SC is the pathogenic agent of contagious bovine pleuropneumonia (CBPP), the most important disease of cattle in Africa causing significant economic losses. The re-emergence of CBPP in Europe in the 1980s and 1990s illustrates that it is still a threat also to countries that have successfully eradicated the disease in the past. Nowadays, probe-based real-time PCR techniques are among the most advanced tools for a reliable identification and a sensitive detection of many pathogens, but only few protocols have been published so far for CBPP diagnosis. Therefore we developed a novel TaqMan^®^-based real-time PCR assay comprising the amplification of two independent targets (MSC_0136 and MSC_1046) and an internal exogenous amplification control in a multiplex reaction and evaluated its diagnostic performance with clinical samples.

**Results:**

The assays detected 49 *Mmm*SC strains from diverse temporal and geographical origin, but did not amplify DNA from 82 isolates of 20 non-target species confirming a specificity of 100%. The detection limit was determined to be 10 fg DNA per reaction for the MSC_0136 assay and 100 fg per reaction for the *MSC_1046 *assay corresponding to 8 and 80 genome equivalents, respectively. The diagnostic performance of the assay was evaluated with clinical samples from 19 experimentally infected cattle and from 20 cattle without CBPP and compared to those of cultivation and a conventional PCR protocol. The two rt-PCR tests proved to be the most sensitive methods and identified all 19 infected animals. The different sample types used were not equally suitable for *Mmm*SC detection. While 94.7% of lung samples from the infected cohort were positively tested in the MSC_0136 assay, only 81% of pulmonal lymph nodes, 31% of mediastinal lymph nodes and 25% of pleural fluid samples gave a positive result.

**Conclusions:**

The developed multiplex rt-PCR assay is recommended as an efficient tool for rapid confirmation of a presumptive CBPP diagnosis in a well-equipped laboratory environment.

## Background

Contagious bovine pleuropneumonia (CBPP) caused by *Mycoplasma mycoides subsp. mycoides *SC (*Mmm*SC) is a disease of cattle affecting the respiratory tract of animals. It is manifested by fever, anorexia, cough, and dyspnoea. Macropathological examinations show gross lesions in the lung including marbling, thickened interlobular septa and sequestra. However, clinical and pathological signs are not always evident, and chronically infected animals might act as carriers and source of infections. Today the disease is mainly confined to sub-Saharan Africa where it causes severe economic losses. As a result of rigid control measures and a consequent stamping out policy, Europe has been CBPP free since the late 1990s. Even so, the risk of re-introducing CBPP remains high. In case of a CBPP outbreak in non-endemic regions caused by animals with inapparent infection or by re- introduction, rapid action such as setting up exclusion zones and culling of affected live stock is essential to efficiently prevent transmission and spread of disease. All these measures depend on an early identification of the pathogen. Two serological tests, the complement fixation test (CFT) and a competitive enzyme-linked immunosorbent assay (cELISA), are approved by the World Organization for Animal Health (OIE) [1], but they are recommended at herd level only [2-5]. Traditionally, mycoplasmas including *Mmm*SC are isolated by culture and identified by biochemical and antigenic techniques [6-8]. Drawbacks of these methods include low sensitivity caused by bacterial contamination, low specificity due to cross-reactivity of antigenic determinants of closely related species and time- and labour-intensive laboratory procedures. Considerable improvements have been achieved by the introduction of PCR since 1994 [2,9,10] which provides a much quicker and more sensitive diagnosis of CBPP. However, due to the close phylogenetic relatedness among *Mmm*SC and other members of the *M. mycoides *cluster including *M. mycoides *subsp. *capri*, M. *capricolum *subsp. *capricolum, M. capricolum *subsp. *capripneumoniae *and *M. leachii*, identification by conventional PCR protocols remains problematic [7]. Real time PCR (rt-PCR) formats using SYBR green detection of PCR products compensated some of the disadvantages connected to conventional PCR [11,12], but are less specific than real-time PCR assays that include specific probes [13,14].

In this study, we developed a new multiplex rt-PCR assay using TaqMan^®^-labelled locked nucleic acid (LNA) probes for the detection of *Mmm*SC and validated it using samples from experimentally infected Boran cattle taken at necropsy. The assay specifically targets the gene *lpp*Q (MSC_1046) and a gene encoding an uncharacterized lipoprotein (MSC_0136) close to the *fba *locus, and it includes an internal amplification control. The new protocol is intended for confirmatory diagnosis of clinically or pathologically suspected cases and is considered to contribute to further improvement of CBPP control in routine laboratory diagnosis.

## Methods

### Bacterial strains

The specificity of the assay was verified by testing DNA from 48 *Mmm*SC field strains, from 46 other type and field strains of the *M. mycoides *cluster to exclude potential cross-reactivity due to close phylogenetic relatedness and from 36 isolates of 14 other relevant mollicute species occurring in cattle (Table 1). All mycoplasma strains were recovered from lyophilized stocks and cultivated in modified Hayflick medium [15] containing 20% horse serum at 37°C for 2-6 days. DNA extracts from the non-related bacterial pathogens *Pasteurella multocida and Mannheimia haemolytica*, that cause respiratory diseases in cattle, were also included in the specificity testing (Table 1). 10 ng of genomic DNA extracted from bacterial cultures were used in the specificity tests.

**Table 1:** Specificity testing of rt-PCR assays with isolates of the *M.mycoides cluster *(A), mollicute strains frequently isolated from bovine lungs (B) and bacterial strains causing CBPP-like symptoms (C)

	Species	Strain	n	Host	Origin	Year	MSC_0136 rt-PCR	MSC_1046 rt-PCR
**A**	***M. mycoides ***	**PG1^T^**		**cattle**	**?**	**?**	**+**	**+**
	**subsp. *Mycoides *SC**	diverse	19	cattle	Italy	1990-1992	+	+
		diverse	3	cattle	Spain	unknown	+	+
		P0		cattle	France	1967	+	+
		N6		cattle	Botswana	1996	+	+
		M375		cattle	Botswana	1995	+	+
		Afade		cattle	Cameroon	1968	+	+
		PO2		cattle	France	1980	+	+
		L2		cattle	Italy	1993	+	+
		Fatick		cattle	Senegal	1968	+	+
		C11		cattle	Chad	1962	+	+
		95014		cattle	Tanzania	1995	+	+
		T1/44		cattle	Tanzania	1952	+	+
		Gladysdale		cattle	Australia	1953	+	+
		Shawawa		cattle	Botswana	1995	+	+
		2091		cattle	France	1984	+	+
		197		cattle	Italy	1992	+	+
		B103		cattle	Portugal	1986	+	+
		6305		cattle	Portugal	1993	+	+
		6526		cattle	Portugal	1993	+	+
		Astercous		cattle	Spain	1987	+	+
		Madrid		cattle	Spain	1993	+	+
		Tan8		cattle	Tanzania	1998	+	+
		Matapi		cattle	Namibia	2004	+	+
		Mandigwan		cattle	Namibia	2001	+	+
		V5		cattle	Australia	1936	+	+
		IS31		cattle	Tanzania	1998	+	+
		2022		cattle	France	1984	+	+
		B237		cattle	Kenya	1987	+	+
	***M. mycoides ***	**PG3, NCTC 10137^T^**		**goat**	**Turkey**	**1950**	**-**	**-**
	**subsp. *capri***	Y-Goat		goat	Australia	1956	-	-
		7302		goat	Portugal	< 1994	-	-
		Kombolcha		goat	Ethiopia	1975	-	-
		7730		goat	France	< 1997	-	-
		9096-C9415		goat	Nigeria	unknown	-	-
		diverse	6	goat	Gran Canaria	1993	-	-
		My-I		goat	Croatia	1986	-	-
		D2482		goat	Switzerland	1991	-	-
		95010		goat	France	1995	-	-
		6443-90		goat	France	1990	-	-
		My-325		goat	Croatia	1986	-	-
		C260/4		goat	Gran Canaria	1993	-	-
		Wk354		goat	Switzerland	1980	-	-
		L		goat	France	1975	-	-
		Wi18079		goat	Germany	2009	-	-
		diverse	3	Barbary sheep	Germany	1994	-	-
		M-5		goat	Croatia	1988	-	-
		M-18		goat	Croatia	1988	-	-
		M-29		goat	Croatia	1988	-	-
								
	***M. capricolum ***	**Cal. kid, NCTC 10154^T^**		**goat**	**USA**	**1954**	**-**	**-**
	**subsp. *capricolum***	C47		sheep	Germany	< 1992	-	-
		4146		goat	France	1980	-	-
		7714		goat	France	1967	-	-
		609-79		goat	France	1979	-	-
		8086-1		goat	France	1980	-	-
	***M. capricolum ***	**F38, NCTC 10192^T^**		**goat**	**Kenya**	**1976**	**-**	**-**
	**subsp. *capripneumoniae***	87F05		goat	Turkey	2005	-	-
		diverse	2	gazelle	UAE	2004	-	-
		3535		mouflon	Qatar	2005	-	-
		GL97P		goat	Tunisia	1980	-	-
	***M. leachii***	**PG50, NCTC 10133^T^**		**cattle**	**Australia**	**1963**	**-**	**-**
		D318b		cattle	Germany	unknown	-	-
		D424		cattle	Germany	unknown	-	-
		Calf1		cattle	Nigeria	unknown	-	-
	*M.nov.spec*.	8756-13		mountain goat	USA	1987	-	-
		G1650		capricorn	Germany	1994	-	-
		G5847		capricorn	Germany	1993	-	-

**B**	***M. putrefaciens***	**KS1, NCTC 10155^T^**		**goat**	**USA**	**1955**	**-**	**-**
		7578.94		goat	France	1994	-	-
		Tours2		goat	France	1972	-	-
	***M. bovis***	**PG45, NCTC 10131^T^**		**cattle**	**USA**	**1962**	**-**	**-**
		363/88		cattle	Germany	1988	-	-
		334/96		cattle	Germany	1996	-	-
		54/10		cattle	Germany	2010	-	-
	***M. bovirhinis***	**PG43, NCTC 10118^T^**		**cattle**	**UK**	**1967**	**-**	**-**
		320/93		cattle	Germany	1993	-	-
		19/94		cattle	Germany	1994	-	-
		365/09		cattle	Germany	2009	-	-
	***M. bovigenitalium***	**PG11, NCTC 10122^T^**		**cattle**	**UK**	**1947**	**-**	**-**
		338/87		cattle	Germany	1987	-	-
		18/92		cattle	Germany	1992	-	-
		466/94		cattle	Germany	1994	-	-
	***M. arginini***	**G230, NCTC 10129^T^**		**mouse**	**USA**	**1968**	**-**	**-**
		162/81		cattle	Germany	1981	-	-
		208/94		Barbary sheep	Germany	1994	-	-
		23/05		sheep	Germany	2005	-	-
	***M. californicum***	**St-6, NCTC 10189^T^**		**cattle**	**USA**	**1981**	**-**	**-**
		456/83		cattle	Germany	1983	-	-
		111/84		cattle	Germany	1984	-	-
	***M. canadense***	**275C, NCTC 10152^T^**		**cattle**	**Canada**	**1974**	**-**	**-**
		380/94		cattle	Germany	1994	-	-
	***M. verecundum***	**107, NCTC 10145^T^**		**cattle**	**UK**	**1974**	**-**	**-**
		49/94		cattle	Germany	1994	-	-
	***M. alkalescense***	**PG51, NCTC 10135^T^**		**cattle**	**Australia**	**1961**	**-**	**-**
		26/93		cattle	Germany	1993	-	-
	***M. canis***	**PG14, NCTC 10146^T^**		**dog**	**UK**	**1951**	**-**	**-**
		306/93		dog	Germany	1993	-	-
	***A. laidlawii***	**PG8, NCTC 10116^T^**		unknown	**UK**	**1967**	**-**	**-**
		54/99		cattle	UK	1999	-	-
	***A. axanthum***	**S743, NCTC 10138^T^**		**tissue culture**	**UK**	**1970**	**-**	**-**
		824/80		cattle	Germany	1980	-	-
	***Ureaplasma diversum***	**A417, NCTC 10182^T^**		**cattle**	**UK**	**1972**	**-**	**-**

**C**	***Pasteurella multocida***	**ATCC 43137^T^**		**pig**			**-**	**-**
	***Mannheimia haemolytica***	**ATCC 33396^T^**		**unknown**			**-**	**-**

### Clinical samples

Tissue samples from an animal infection trial performed according to the Kenyan national legislation for animal experimentation and approved by the ILRI Institutional Animal Care and Use Committee (IACUC reference number 2008.08) were obtained from the International Livestock Research Institute Nairobi, Kenya [16]. Twenty 14-16 months old castrated outbred Boran cattle (*Bos indicus*) were infected intratracheally with 5 × 10^10 ^cfu of the strain *Mmm*SC Afade in 50 ml culture broth. Six days post infection nine animals were depleted for CD4+ T lymphocytes using repeated injections of CD4-specific mouse monoclonal antibody. The CD4+ T cell depletion had no discernable effect on the infection status, clinical presentation, pathology or humoral response of the animals [16]. Out of the 20 infected cattle, 19 were available for sampling. Three of them (animals no. BD097, BD098, BD118) showed acute CBPP-typical clinical signs such as coughing, dyspnea and fever (> 39.4°C) for at least eight consecutive days. Because of the severity of the disease, these animals had to be euthanized 16 to 21 days post infection. Out of the 16 surviving animals, 13 showed clinical signs, and three animals (no. BD096, BD100, BD111) were asymptomatic. All were euthanized 28-30 days post infection.

Post mortem examination of lung tissue revealed typical pathological signs of pneumonia and respiratory disorder in all but one (no. BD102) individuals. Gross lesions of red hepatisation and marmorisation, necrosis and sequestra were seen in animals no. BD097, BD098 and BD118, whereas fibrous adhesions between pleura and chest wall and the production of massive amounts of pleural fluid (a typical symptom of acute CBPP) were only observed in animals no. BD097 and BD118. All other animals, with the exception of no. BD102, developed necrotic lesions, sequestra and areas of resolution of differing sizes. Their low morbidity together with the appearance of lung lesions indicated that these animals were in the chronic stage of infection. Despite the artificial infection and the constant contact with its infected companions, animal no. BD102 did not show any pathological changes in the lung, and also clinical signs were mild. All animals showed a specific antibody response detected by the complement fixation test (CFT) (data not shown). According to the case definition for CBPP given by the OIE [17], 18 animals are defined as *Mmm*SC-positive and animal no. BD102 as *Mmm*SC-negative. But since animal no. BD102 had *Mmm*SC specific antibodies and mild clinical symptoms (fever) it was also included in the positive group.

Specimens of lungs and lymph nodes as well as pleural fluid were collected, immediately frozen at -80°C and used for DNA extraction and subsequent PCR.

Re-isolation of *Mmm*SC was done by inoculation of 50-100 mg smashed tissue or 300 μl pleural fluid into 3 ml modified Hayflick broth containing 20% horse serum and 1000 IU/ml penicillin for 3-5 days. Subsequently, an aliquot was plated onto a modified Hayflick agar plate without penicillin to control for growth of mycoplasma colonies. Sub-cultures were grown in 3 ml of modified Hayflick broth without penicillin to identify the re-isolated species. DNA was extracted and conventional *Mmm*SC-specific PCR was performed as described below.

20 negative control samples from cattle flocks in Germany (which is CBPP-free) were included in the analysis. Lothar Hoffmann (TLLV Bad Langensalza, Germany) kindly provided lung samples of cattle diagnosed with pneumonia (n = 3). Bovine lung samples from an experimental chlamydia infection (approved by the Thuringian State Office for Food Safety and Consumer Protection under registration no. 04-002/07) causing pneumonia in cattle (n = 8) and lung samples from uninfected animals (n = 9) were obtained from the animal facilities of the Friedrich Loeffler Institute.

### DNA extraction

Cells from 3 ml broth cultures were harvested by centrifugation, washed twice in PBS, and DNA was extracted using the High Pure PCR Template Preparation Kit™ (Roche Diagnostics, Mannheim, Germany) according to the manufacturer's instructions. DNA extraction from animal tissue was performed following the protocol of the innuSPEED Tissue DNA Kit™ (Analytik Jena, Jena, Germany) and DNA was eluted with 100 μl of buffer.

### Confirmation of strain identity

All strains presumably belonging to the *M. mycoides *cluster were characterized by amplification and sequencing of a 992 bp fragment of *uvr*C.

*M. mycoides *cluster strains other than *Mmm*SC were additionally analyzed by amplification and sequencing of fragments of *rpo*B. Universal primers *uvr*C-F1 5'-GATCTATTTTATTAACACTACAACG-3' and *uvr*C-R2 5'-TCTTTAGCTGCATGAATTTG and universal *rpo*B-specific primers as described by Manso-Silvan et al. and Vilei et al. [18,19] were used at a concentration of 400 nM in a 25 ul PCR reaction volume with 1.5 mM MgCl_2_, 250 μM each dNTP, reaction buffer and 1 U InnuTaq Hot A DNA Polymerase (Analytik Jena, Jena, Germany). The temperature-time profile on a UNO II thermocycler (Biometra, Göttingen, Germany) was 95°C for 5 min and 35 times 95°C for 30 s, 50°C for 30s and 72°C for 60 s. Amplicons were purified by the DNA Clean and Concentrator Kit (Zymo Research, Irvine, USA) and subsequently sequenced by Eurofins MWG Operon (Ebersberg, Germany) using the respective forward primers. Sequences were edited by the software BioEdit™ version 7.0.9 and aligned and compared to reference sequences by the software tool Multalin™ [20].

The field strains of other mycoplasma species of bovine origin investigated in this study were isolated in our lab over the last decades and identified by an in-house indirect immunofluorescence test according to Rosendal and Black, [8].

### Conventional *Mmm*SC-specific PCR

Conventional PCR [10] was done on dilution series of quantified DNA from cultures and DNA extracted from clinical specimens using the primer pair SC3NEST1-L/SC3NEST1-R and Taq DNA Polymerase 5 Prime (VWR International, Darmstadt, Germany) on a UNO II thermocycler. 5 μl of each PCR product were analyzed by gelelectrophoresis and visualised by ethidiumbromide staining.

### Oligonucleotide Primers and TaqMan^® ^probes

*Mmm*SC-specific primers and probes were designed using the software tool IDT OligoAnalyzer3.1 based on sequence comparisons between the respective genes of *Mmm*SC field strains and type and field strains of the *M. mycoides *cluster performed by the software tool Multalin™ [20].

Locked nucleic acid (LNA)-containing dual-labelled probes, designed in cooperation with and produced by TIB Molbiol (Berlin, Germany), were applied to compensate the low GC-content of mycoplasma DNA and to ensure specific and sensitive probe hybridization despite the low Tm values of probe sequences.

The primers MSC_1046-S and MSC_1046-R2 obtained from MWG Operon (Ebersbach, Germany) (Table 2) target the gene of a well-characterized antigenic and specific *Mmm*SC lipoprotein [21]. This gene occurs in two copies in type strain PG1, but only in one copy in many other *Mmm*SC strains [22]. The respective probe MSC_1046-TM1 was designed to anneal to a highly *Mmm*SC-specific segment exhibiting ten nucleotide mismatches to the homologous sequences from *M. mycoides *subsp. *capri *(GenBank accession number FQ377874) and *M. leachii *(CP002108).

**Table 2:** Primers and TaqMan^® ^probes

Target	Primers/Probes	Sequence (5'-3')	Position^2^
IppQ MSC_0136 IAC	MSC_1046-S MSC_1046-R2 MSC_1046-TM1 MSC_0136-F MSC_0136-R MSC_0136-TM EGFP-1F EGFP-10R EGFP-Hex	ATCAAGATATTTCGAGTTGAAATGTAAG TGTATATTTTTTAGATTTCAATCTGAAAGTG **FAM**-TTTCAGCTCGATAAAACATATTT-**BBQ^1^** AACACAAAAAACCGAACAGCCT AGCTTATCAGGAACTTTTTTAACGTA **Cy5**-TGAGATAGTTCAAATTGGCTTT-**BBQ^1^** GACCACTACCAGCAGAACAC CTTGTACAGCTCGTCCATGC **HEX**-AGCACCCAGTCCGCCCTGAGCA-**BHQ1**	1166686-1166713 1190443-1190470 1166797-1166767 1190554-1190524 1166754-1166732 1190511-1190489 159994-160015 1023331-1023310 160131-160106 1023194-1023219 160042-160063 1023283-1023262

^1^Locked nucleic acid (LNA) containing nucleotides are underlined,^2^Based on GenBank sequence BX293980

Primers MSC_0136-F and MSC_0136-R mediate the amplification of the target MSC_0136, which codes for a hypothetical transmembrane protein and is located directly adjacent upstream of the *fba *locus [23]. The PG1 genome also harbours a second copy of the gene in reverse orientation [24]. Amplification products were detected by hybridization and hydrolysis of probe MSC_0136-TM which was designed with at least 3 mismatches to sequences from other members of the *M. mycoides *cluster.

### Internal amplification control

A 712 bp fragment of the enhanced green fluorescent protein (EGFP) gene was used as internal amplification control (IAC) template as described previously [25] which is also commercially available as Intype IC-DNA (Labor Diagnostik, Leipzig, Germany). A 177 bp amplicon was generated by amplification with primers EGFP-1F and EGFP-10R and detected by probe EGFP-HEX to demonstrate the absence of PCR inhibitory substances.

### Multiplex real-time PCR

Amplifications were performed as duplex assays (MSC_0136 and MSC_1046 assay) with culture material and as triplex assays including also the IAC with tissue samples. The assays were run on a Mx3000P^® ^thermocycler (Stratagene, Heidelberg, Germany) using the following cycling parameters: initial denaturation at 95°C for 5 min, 45 cycles of denaturation at 95°C for 20 s, primer annealing at 57°C for 45 s and extension at 68°C for 45 S. Optimal primer and probe concentrations were determined by empirical testing of different concentrations ranging from 200 nM to 800 nM final concentration. Finally, each 25 μl duplex reaction contained 12.5 μl 2 × QuantiTect^® ^Multiplex PCR Master Mix (Qiagen, Hilden, Germany), 400 nM each of primers MSC_1046-S, MSC_1046-R2, MSC_0136-F and MSC_0136-R, 300 nM each of probes MSC_1046-TM1 and MSC_0136-TM and 1 μl of DNA template. Additionally, 800 nM of the EGFP-specific primers and 400 nM of probe EGFP-HEX as well as 0.25 μl IAC template DNA corresponding to 12,500 copies were added to reactions in the triplex assay.

Amplified products were analyzed using the Stratagene software MxPro™, and amplification plots were generated with an adaptive baseline. The threshold, i.e. the level of fluorescence signal that reflects a significant increase over the baseline signal, was manually adjusted at 0.2 for the MSC_0136 assay and at 0.15 for the MSC_1046 assay. The threshold cycles were calculated accordingly by the instrument.

Each sample was analysed by rt-PCR in duplicate. Each microtitre plate included a serial dilution of quantified standard DNA containing 10 fg to 10 ng.

### Standard curve calculation

Standard curves were generated from duplicates of five tenfold dilution series of *Mmm*SC PG1 genomic DNA quantified by UV-spectrophotometry (Nanodrop^®^, Thermo Scientific, Wilmington, USA) containing 1 fg - 100 ng. Linear regressions, efficiencies and correlation coefficients were calculated from the standard graphs of the Ct values plotted against the logarithm of the DNA amount, and intra-assay variability was determined. Additionally, five independent runs on different days were performed in duplicates to assess inter-assay variance and coefficients of variance were calculated.

### Correlation of cfu and DNA content

To investigate the correlation between counts of colony forming units (cfu) and extracted DNA and to define an adequate reference value for pathogen quantification, a growth curve of *Mmm*SC PG1 was recorded. Cfu counts and the respective quantities of total DNA determined using the newly developed rt-PCR assay were compared at different time points. Two-day cultures of *Mmm*SC were diluted 1:100 into fresh liquid medium and incubated at 37°C. Every 6 to 12 h up to 48 h, 100 μl aliquots were removed, tenfold serial dilutions were prepared and 20 μl of each dilution were seeded on agar plates for counting cfus after incubation of the plates for 3 days. The doubling time of the strain was calculated during the logarithmic growth phase with Doubling Time Software v1.0.10 (Roth, 2006, http://www.doubling-time.com/compute.php).

Furthermore, aliquots were taken after 12, 18, 24 and 48 h of growth from which tenfold dilution series were prepared. DNA was extracted from 200 μl of each dilution sample as described above and quantified in duplex rt-PCR assays. Results were compared with standard curves obtained from tenfold serial dilutions of quantified *Mmm*SC DNA. The calculated genome equivalents of the 10^-1 ^dilution samples at the time points 12, 18, 24 and 48 h were related to the respective cfu counts.

## Results

### Specificity testing

In order to determine the ability of the MSC_1046 and MSC_0136 assays to detect *Mmm*SC DNA, genomic DNA extracts of the type strain PG1 and of 48 *Mmm*SC field strains were subjected to the duplex rt-PCR. All isolates were readily detected by both assays.

All non-*Mmm*SC strains belonging to the *M. mycoides *cluster (46 strains) and 36 isolates of 16 other mollicute and bacterial species yielded a signal below a Ct value of 39 in any of the two amplification reactions. The results are summarized in Table 1 and confirm an analytical specificity of the developed duplex rt-PCR of 100%.

### Detection limit and standard curves of MSC_1046 and MSC_0136 assay

**Figure 1:**
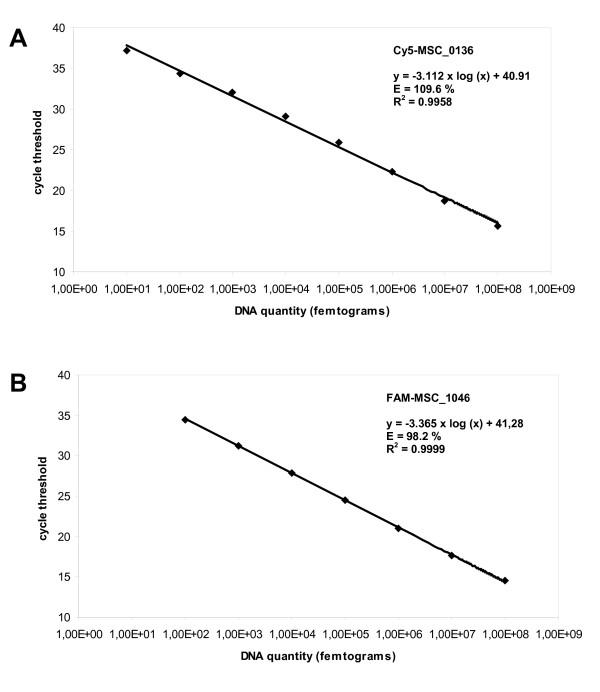
**Real-time quantification of DNA extracted from *Mmm*SC cultures**. DNA concentration was measured spectrophotometrically and adjusted to 100 ng μl^-1^. Five tenfold dilution series were prepared and applied in duplex rt-PCR assays comprising the amplification and detection of the targets MSC_0136 (A) and MSC_1046 (B) with the TaqMan^® ^LNA probes FAM-MSC_1046 and Cy5-MSC_0136, respectively. The detection limits are 10 fg DNA and 100 fg DNA corresponding to approximately 8 genome equivalents and 80 genome equivalents for the MSC_0136 and MSC_1046 assay, respectively.

Five tenfold serial dilutions of genomic DNA covering a range from 1 fg to 100 ng were analysed in duplex assay to calculate detection limits (LOD) and standard curves for MSC_1046 and MSC_0136 amplifications (Figure 1). The LOD giving a reproducible signal (10 out of 10) was determined to be 10 fg DNA per reaction for the MSC_0136 assay and 100 fg per reaction for the MSC_1046 assay corresponding to 8 and 80 genome equivalents, respectively, based on the size of the published *Mmm*SC genome sequence [24]. Conventional PCR performed with the same dilution series exhibited a detection limit of 1 pg, corresponding to 800 genome equivalents and thus, proved to be less sensitive by an order of 1-2 log units.

While the standard curve of the MSC_1046 reaction is linear over a range of 7 log units (from 100 fg to 100 ng), as illustrated by a correlation coefficient (R^2^) of 0.9999, the standard curve of the MSC_0136 reaction shows linearity over a range of 6 log units (from 1 pg to 100 ng) only and has a lower R^2 ^value of 0.9958 for the entire detectable dilution range of 8 log units. The efficiency of amplification calculated from standard curve slopes was determined by the instruments software to be 109.6% for MSC_0136 and 98.2% for MSC_1046. Intra-assay variation coefficients varied between 1.1 and 2.6% for MSC_0136 and between 0.7 and 2.3% for MSC_1046. Inter-assay variation coefficients ranged from 1.1 to 2.4% for MSC_0136 and from 0.8 to 2.9% for MSC_1046 indicating a good reproducibility also between different runs.

When compared to the two simplex reactions, the duplex format did not shift Ct values and did in particular not compromise the sensitivity of the two assays. Also the presence of the IAC did not negatively influence the amplification of *Mmm*SC DNA in triplex rt-PCR (data not shown).

### Growth curve and *Mmm*SC quantification

**Figure 2:**
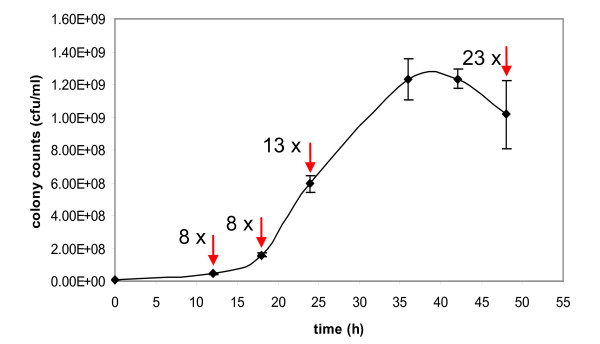
**Growth curve of *Mycoplasma mycoides *subsp. *mycoides *SC PG1 expressed in colony forming units (cfu/ml)**. Numbers next to arrows indicate factors by which colony counts differ from genome equivalents calculated after analysis of extracted DNA in the presented MSC_1046 rt-PCR assay.

The growth curve in Figure 2 was obtained for *Mmm*SC type strain PG1 after colony counting under cultivation conditions described above. The culture reached the stationary phase after 24 to 36 hours of cultivation. The doubling time during the logarithmic phase after 18 to 24 hours of cultivation was 188 min which is in the range reported for *Mmm*SC strains [26]. In parallel, cultures at time points 12, 18, 24 and 48 h were examined through the quantification of extracted DNA by MSC_1046 rt-PCR assay. As expected, the total amount of deduced genome equivalents exceeded the number of viable mycoplasmas determined by colony counting. While in the lag phase (12 h) and early logarithmic phase (18 h), the total amount of DNA expressed in genome equivalents exceeded the cfu values by a factor of 8, this factor rose to 13 in the culture after 24 h of growth and to 23 after 48 h.

### Detection of *Mmm*SC in clinical samples

Clinical samples were subjected to the triplex rt-PCR assay comprising the amplification of the two *Mmm*SC-specific targets MSC_0136 and MSC_1046 and the IAC template. The IAC template was consistently amplified in all samples as illustrated by a Ct value of approximately 27 ruling out a PCR inhibition in the sample preparations.

Lung samples from cattle flocks in Germany with and without diagnosed pneumonia were used as negative control samples and examined in the multiplex rt-PCR and conventional PCR. All negative control samples yielded negative results as expected.

66 clinical specimens obtained from 19 animals of the CBPP infection trial were investigated by the multiplex rt-PCR, conventional PCR and cultivation (Table 3). 19 samples were positive in all tests, whereas 23 samples yielded negative results in all tests. 30 specimens (45.5%) were culture positive, but 41 samples (62.1%) were tested positive with the MSC_0136 assay and 37 samples (56.1%) with the MSC_1046 assay. 19 samples (28.8%) gave a positive result with the conventional SC3 PCR.

**Table 3:** Detection of *Mycoplasma mycoides *subsp. *mycoides* SC in lung, lymph nodes and pleural fluid from experimentally infected Boran cattle by cultivation, MSC_0136 and MSC_1046 rt-PCR and conventional SC3-PCR assay

Animal	Lung				Lymph nodes, pulmonal				Lymph nodes, mediastinal				Pleural fluid			
	
	Culture	rt-PCR		PCR	Culture	rt-PCR		PCR	Culture	rt-PCR		PCR	Culture	rt-PCR		PCR
		MSC 0136	MSC 1046	SC3		MSC 0136	MSC 1046	SC3		MSC 0136	MSC 1046	SC3		MSC 0136	MSC 1046	SC3
BD091	+	+	+	+	n.d.	n.d.	n.d.	n.d.	n.d.	n.d.	n.d.	n.d.	n.d.	n.d.	n.d.	n.d.
BD092	+	+	+	-	-	+	+	-	+	-	-	-	-	-	-	-
BD093	+	+	+	+	-	+	-	-	+	+	-	-	-	-	-	-
BD094	+	+	+	+	-	+	+	-	-	-	-	-	-	-	-	-
BD095	+	+	+	+	+	-	-	-	-	-	-	-	-	-	-	-
BD096	-	+	+	+	-	-	-	-	-	-	-	-	-	-	-	-
BD097	+	+	+	+	n.d.	n.d.	n.d.	n.d.	n.d.	n.d.	n.d.	n.d.	+	+	+	+
BD099	+	+	+	-	-	-	-	-	-	-	-	-	-	-	-	-
BD100	+	+	+	+	-	+	-	-	-	-	-	-	-	-	-	-
BD101	+	+	+	+	+	+	+	-	+	+	-	-	-	+	+	-
BD102	-	-	-	-	-	+	+	-	-	-	-	-	-	+	+	-
BD105	+	+	+	+	-	+	+	-	n.d.	n.d.	n.d.	n.d.	-	-	-	-
BD106	-	+	+	-	+	+	+	-	-	-	-	-	-	-	-	-
BD107	+	+	+	+	+	+	+	-	-	+	+	-	-	-	-	-
BD111	+	+	+	+	+	+	+	-	-	-	-	-	n.d.	n.d.	n.d.	n.d.
BD115	+	+	+	+	-	+	+	-	-	+	+	-	n.d.	n.d.	n.d.	n.d.
BD116	+	+	+	+	+	+	+	+	+	+	+	-	-	-	-	-
BD118	+	+	+	+	n.d.	n.d.	n.d.	n.d.	n.d.	n.d.	n.d.	n.d.	+	+	+	+
BD119	+	+	+	+	+	+	+	+	+	+	+	-	-	-	-	-
positive/total	16/19	18/19	18/19	15/19	7/16	13/16	11/16	2/16	5/15	6/15	4/15	0/15	2/16	4/16	4/16	2/16

n.d.: not done, +: positive test, -: negative test, rt: real time.

All infected animals were correctly identified by the rt-PCR assays if results from different sample types were considered. Thus, calculation of test sensitivities based on diagnosis for individual animals resulted in sensitivities of 100% for the MSC_0136 and MSC_1046 rt-PCR, 78.9% for the SC3 PCR and 89.5% for culture (Table 4).

**Table 4:** Detection rates of *Mycoplasma mycoides *subsp. *mycoides *SC in CBPP-infected animals after parallel investigation of lung, lymph nodes and pleural fluid by cultivation, MSC_0136 and MSC_1046 rt-PCR and conventional SC3-PCR assay

Animal: n = 19, Condition: positive = 19				
		**rt-PCR**		**PCR**
	**Culture**	**MSC_0136**	**MSC_1046**	**SC3**

Positives	17	19	19	15
Detection rate	89.5%	100%	100%	78.9%

n = total number, rt: real time.

Table 5 presents test specificities and sensitivities based on data derived from the examination of lung specimens. No false positive results were obtained in any test resulting in specificities of 100% for the PCR assays. The two rt-PCR assays failed to identify one out of 19 lung samples as *Mmm*SC-positive (sensitivity of 94,7%), but are more sensitive than detection by culture (84. 2%) and conventional SC3 PCR (78.9%).

**Table 5:** Comparison of specificity and sensitivity of different tests for the detection of *Mycoplasma mycoides *subsp. *mycoides *SC in lung tissue

Lung: n = 39, Condition: positive = 19, negative = 20				
		**rt-PCR**		**PCR**
	**Culture**	**MSC_0136**	**MSC_1046**	**SC3**

True positives	16	18	18	15
False positives	0	0	0	0
False negatives	3	1	1	4
True negatives	**-**	20	20	20
Sensitivity	84.2%	94.7%	94.7%	78.9%
Specificity	-	100%	100%	100%

n = total number, rt: real time.

A strong difference was observed in rt-PCR test results obtained from lung samples, lymph nodes and pleural fluid samples (Table 3). While 94.7% of lung samples were positively tested in the MSC_0136 assay, 81% of pulmonal lymph nodes, 31% of mediastinal lymph nodes and 25% of pleural fluid samples gave a positive result.

## Discussion

Sensitive, reliable and time-saving molecular diagnostic methods are indispensible tools for a successful control of contagious bovine pleuropneumonia. Here, we present the development and preliminary evaluation of a new TaqMan^®^-based real-time PCR assay for the detection of the CBPP agent *Mycoplasma mycoides *subsp. *mycoides *SC in clinical samples. The assay comprises the amplification of two independent targets, MSC_1046 and MSC_0136, as well as an internal exogenous amplification control in a multiplex reaction. Nowadays, PCR is an accepted alternative to time- and labour-intensive and often insensitive culture-based methods for the direct detection of *Mmm*SC in infected animals to confirm a presumptive clinical or pathomorphological diagnosis [17]. In general, PCR is known as a sensitive, specific, straightforward and fast tool for pathogen detection and several conventional PCR tests have been proposed to detect *Mmm*SC [2,9,10,27-29]. Despite the superiority of real time-PCR assays over conventional PCR and the importance and impact of the disease, only few rt-PCR protocols have been introduced so far and none of them was validated on more than five clinical samples. Gorton et al. [13] published three independent assays with MGB-TaqMan^® ^probes with rather low amplification efficiencies and detection limits of 100 fg to 1 pg. Higher sensitivities were reached with a SYBR Green^® ^rt-PCR assay [12], but the lack of specific amplicon detection by a probe might negatively influence the specificity of the assay. Recently, Vilei and Frey [14] reported on the development of two assays with TaqMan^® ^probes intended for the detection of *Mmm*SC in bronchoalveolar lavage fluid of cattle and for the discrimination of wild type strains from a mutant vaccine strain. The assays are based on the gene of the well-described antigenic surface lipoprotein LppQ [21,30] that is also used in the study presented here. However, in our study, we preferred LNA probes to Minor Grove Binding (MGB) probes as they were shown to be significantly more sensitive, have a low optimum concentration and low background fluorescence [31] and are also cheaper. Furthermore, we used a probe binding site downstream of the sites proposed by Vilei and Frey [14] which guaranteed a more specific detection due to a higher number of nucleotide mismatches to the homologous sequences from *M. mycoides *subsp. *capri *and *M. leachii*. The differentiation between *Mmm*SC and its close relatives of the *M. mycoides *cluster is important and best performed by the use of variable genetic targets with high discriminatory potential such as MSC_1046 and MSC_0136. In fact, our duplex assay was able to detect DNA of all of the 48 tested *Mmm*SC strains and did not cross-react with any non-target strain indicating excellent specificity. The analytical sensitivity and detection limits of the two single assays were in the range reported for rt-PCR tests. The lower LOD of the MSC_0136 assay compared to the MSC_1046 assay was also reflected in its higher diagnostic sensitivity. Although both assays are predominantly designed for qualitative pathogen detection, especially the MSC_1046 assay with a dynamic range of 7 log units may also be a useful tool for quantitative evaluation of pathogen load. Standard curve preparation and pathogen quantification by rt-PCR are often related to cfu counts assuming a strict correlation between numbers of viable cfu and extracted DNA [12,14]. We have tested this assumption and found that the total amount of detected DNA exceeded the counted cfu already in the lag and early exponential phase by eight times and that the ratio varies depending on the age of the culture (Figure 2). Thus, especially for slow growing fastidious organisms such as mycoplasmas, the use of cfu instead of DNA amount may lead to an overestimation of sensitivity when assessing analytical test performance since the contribution of non-viable genome equivalents to total DNA amount is not considered.

In accordance with recommendations after which the detection of *Mmm*SC by PCR should be performed by the amplification of two independent targets [1,7], our new rt-PCR test was conceived as a multiplex assay including two targets and an internal control without loss of sensitivity. The multiplex format is advantageous over simplex assays as it is particularly economical in terms of cost of material (mastermix, consumables) and turnaround time and suitable for small volume samples and high-throughput.

The clinical usability of a PCR test is not solely dependent on its inherent specificity and sensitivity, but also influenced by the course and stage of infection, sampling strategy and sample preparation. To evaluate the ability of the new test to diagnose *Mmm*SC infection in cattle, different specimens from CBPP infected animals and from cattle with non-*Mmm*SC related pneumonia as well as from healthy animals were examined. It was evident that the extraction procedure efficiently removed DNA polymerase inhibitors from the tissue matrices since amplification of the IAC template was not impaired. However, DNA degradation was observed in some samples after long-time storage at -20°C possibly due to nucleases remaining in the samples after DNA preparation. Therefore, an immediate processing of samples after DNA extraction is advisable.

The rt-PCR assays' diagnostic specificity proved to be 100%. Diagnostic sensitivities were compared to those of *Mmm*SC detection by culture and conventional PCR. The MSC_0136 and MSC_1046 assays were found to be most sensitive. Concerning the diagnosis for the individual animal, the sensitivity was 100% for both rt-PCR assays, compared to 89.5% for culture and 78.9% for SC3 PCR (Table 4). Thus, both rt-PCR assays reliably identified infected animals provided that different clinical specimens were investigated. If lung samples were exclusively considered, diagnostic sensitivity was 94.7% for the two rt-PCR assays, 84.2% for culture and 78.9% for the conventional PCR test (Table 5). In contrast, lymph nodes were obviously less appropriate to detect *Mmm*SC in infected animals. This observation is congruent to findings of a study using tissue samples from a natural CBPP outbreak in Portugal [32]. As expected, pathogen detection was more often successful in pulmonal lymph nodes that directly drain the lungs than in the more distant mediastinal lumph nodes. Pleural fluid was previously recommended as the sample of choice for the diagnosis of CBPP [33]. However, it is not always present in clinical cases of CBPP. We could detect high titres of *MmmSC *(Ct values for MSC_0136 below 23 correlating to > 2.3E+08 GE/ml) in the voluminous pleural fluid samples from the two acutely infected animals no. BD097 and BD118, whereas the other animals accumulated only tiny amounts of pleural fluid of which only two tested positive in the rt-PCR assay with Ct values close to the detection limit (> 36.0). In conclusion, the apparent weak diagnostic performance in the investigation of single specimens has to be attributed to the used sample types being not all equally suitable.

Animal no. BD102 did not show any pathological modifications in the lung, a fact that did not allow targeted sampling. This may explain the negative rt-PCR result obtained for the animal's lung sample. However, *Mmm*SC was detected in lymph node tissue and pleural fluid of the animal, which underlines the importance of a diversified sampling procedure, especially in animals with negative pathology, in order to prevent false negative results [34,35]. The frequently reported cases of detection of *Mmm*SC by PCR in samples of asymptomatic animals without organic lesions and without successful cultivation ([36,34,32], present study) are not reflected by the OIE case definition of CBPP. However, those animals are nevertheless important as carriers and shedders.

Although the number of samples was limited and far from satisfying the Principles of Validation of the OIE Terrestrial Manual [1], the pre-validation of the new rt-PCR assay presented here is a first important step in assessing its diagnostic capabilities. In the future, well-characterized field samples from endemic areas are needed for further validation of the assay according to OIE guidelines.

## Conclusions

1) The developed multiplex rt-PCR is more sensitive than cultivation and conventional PCR. All infected animals could be identified, but only a combined analysis of different sample types ensured 100% sensitivity of the test.

2) Lung samples collected from lesions at the interface between diseased and healthy tissue are most appropriate to detect *Mmm*SC infections and should predominantly be considered in the analysis by rt-PCR. However, if resources allow, we propose a more comprehensive sampling strategy including also lymph node tissue and pleural fluid to clarify the infection status of the animal.

3) In conclusion, we recommend the duplex rt-PCR assay as an efficient tool for rapid confirmation of a presumptive CBPP diagnosis based on clinical suspicion or pathological observations especially in a laboratory setting of developed countries and for epidemic surveillance in CBPP-free countries. In the future, the test will be used in routine diagnosis of CBPP in the German Reference Laboratory.

## Authors' contributions

CS planned and supervised microbiological and preparative laboratory work, designed and conducted the real-time PCR experiments and wrote the manuscript. MH coordinated the project, organized funding and helped in sample acquisition at the animal trial. JJ designed and carried out the animal infection experiment. HT assisted in data analysis and reviewed the manuscript. HN contributed to acquisition of funding, organisation of the project and reviewing the manuscript. All authors have read and approved the final manuscript.
